# The impacts of minimally invasive surgery on intermediate- or high-risk cervical cancer patients received adjuvant radiotherapy

**DOI:** 10.1186/s12957-022-02820-x

**Published:** 2022-11-28

**Authors:** Qiying Zhang, Zi Liu, Yali Wang, Jing Zhang, Wen Li, Tao Wang, Juan Wang, Fan Shi, Jin Su

**Affiliations:** 1 Department of Radiation Oncology, The First Affiliated Hospital of Xi’an Jiaotong University, No.277, West Yanta Road, Xi’an, Shaanxi 710000 People’s Republic of China; 2 Department of Radiation Oncology, The Second Affiliated Hospital of Xi’an Jiao Tong University, Xi’an, 710004 People’s Republic of China

**Keywords:** Cervical cancer, Minimally invasive surgery, Open surgery, Radiotherapy, Chemotherapy

## Abstract

**Background:**

Adjuvant chemoradiotherapy (CRT) has been shown to reduce the risk of recurrence for patients with risk factors after radical hysterectomy (RH). Early initiated CRT could result in superior oncological outcomes. Here, we aimed to compare the survival outcome of intermediate- or high-risk cervical cancer (CC) patients who, received adjuvant CRT between minimally invasive surgery (MIS) and open surgery.

**Methods:**

Data on stage IB1-IIA2 patients who underwent RH and postoperative CRT in our institution, from 2014 to 2017, were retrospectively collected. Patients with high or intermediate-risk factors who met the Sedlis criteria received sequential chemoradiation (SCRT). According to the surgical approaches, the enrolled patients were divided into MIS and open surgery groups. Then, the disease-free survival (DFS), overall survival (OS), and prognostic factors were analyzed.

**Results:**

Among 129 enrolled CC patients, 68 received open surgery and 61 received MIS. The median time interval from surgery to chemotherapy and to radiotherapy was shorter in the MIS group (7 days vs. 8 days, *P*=0.014; 28 days vs. 35, *P*<0.001). Three-year DFS and OS were similar in both groups (85.2% vs. 89.7%, *P*=0.274; 89.9% vs. 98.5%, *P*=0.499). Further, sub-analysis indicated that the DFS and OS in intermediate/high-risk groups had no significant difference. Cox-multivariate analyses found that tumor size >4 cm and time interval from surgery to radiotherapy beyond 7 weeks were adverse independent prognostic factors for DFS.

**Conclusion:**

Based on the population we studied, for early-stage (IB1-IIA2) CC patients with intermediate- or high-risk factors who received postoperative SCRT, although the difference was not significant, the DFS and OS in the MIS group were slightly lower than the ORH group, and tumor size >4 cm and delayed adjuvant radiotherapy beyond 7 weeks were risk factors for recurrence.

**Supplementary Information:**

The online version contains supplementary material available at 10.1186/s12957-022-02820-x.

## Background

Women with early-stage cervical cancer (CC) can be treated with surgery or chemoradiation (CRT), but most opt for surgery [1]. Traditionally, open radical hysterectomy (RH) has been the recommended standard of care, but limited for the complications [2, 3]. Contrastively, minimally invasive surgery (MIS) showed better surgery-related outcomes [4, 5]. Besides, they have comparable 5-year overall survival (OS) and disease-free survival (DFS) rates [6, 7].

However, the publication of the laparoscopic approach to carcinoma of the cervix (LACC) trial changed our vision of the surgical treatment for these women. This trial demonstrated that MIS had a higher recurrence rate and a lower DFS and OS rate than open surgery [8]. Comparable results were also reported by Melamed et al. [9] in their cohort study. Hence, these poor survival outcomes with MIS prompted considerable debate regarding the appropriateness of MIS for CC. However, we cannot completely deny the clinical benefits of MIS, and now, MIS is still widely used in other tumors. Therefore, it is necessary to further study the impact of surgical methods on survival benefit and also to explore the mechanism of its negative impact on survival and the way to eliminate the negative impact.

Hence, in this study, we analyzed the survival outcomes of early-stage CC patients with intermediate- or high-risk factors who received radiotherapy (RT) or CRT following MIS or open surgery at our institution. Besides, we also investigated prognostic factors that might affect survival outcomes in those patients.

## Methods

### Patients

This retrospective study included CC patients diagnosed with stage IB1-IIA2 according to the 2009 FIGO staging system [10] between 2014 and 2017. Based on the surgical approach, the patients were divided into two groups: the open surgery group and MIS group. The study was approved by the Ethics Committee of the First Affiliated Hospital of Xi’an Jiaotong University (No. XJTU1AF2021LSK-257). The informed consent was exempted due to the retrospective nature of the study.

The inclusion criteria were (1) all patients underwent a type III RH with pelvic lymph node dissection [11]; (2) patients with one or more high-risk factors (positive lymph nodes, parametrial involvement, and positive margin status) were categorized into high-risk prognostic group [12]; (3) patients negative for any of the high-risk factors and positive for combined intermediate-risk factors (lymph vascular space invasion (LVSI), stromal invasion and tumor size) that meet the Sedlis criteria were categorized into intermediate-risk prognostic group [13]; and (4) received postoperative external beam radiotherapy (EBRT), with or without chemotherapy (CT). The exclusion criteria were (1) patient previously received RT, (2) patients with absence of clinical or pathological data, and (3) patient relapsed before receiving RT.

### Treatment strategies

Enrolled patients received open surgery or MIS. Following surgery, postoperative adjuvant therapy is recommended for patients with intermediate- or high-risk factors according to the NCCN guidelines [14]. For patients with pathological high-risk factors should receive adjuvant RT combined with platinum-based CT (4–6 cycles). For patients with combined intermediate-risk factors that meet the Sedlis criteria received adjuvant RT and 2–4 cycles of platinum-based CT. Due to the limited radiation resource, postoperative adjuvant therapy was performed by sequential chemoradiation (SCRT), which was similar with the SCRT arm published by Huang et al. [15]. Platinum-based chemotherapy was given 1–2 cycles prior to and 2–4 cycles after radiotherapy, respectively, except for patients who are allergic to, cannot tolerate, or refuse chemotherapy. Patients generally started radiation within 6 weeks after surgery, with some patients delayed due to urinary retention, infection, and poor wound healing. The radiotherapy technology used for EBRT was a three-dimensional conformal radiation therapy (3D-CRT) before 2015 and intensity-modulated radiotherapy (IMRT) after that, combined with computed tomography-based treatment planning. The clinical target volume was determined using the Radiation Therapy Oncology Group criteria [16]. EBRT was delivered at a dose of 2 Gy/d 5 days per week (total dose 50 Gy). Brachytherapy was given to patients with positive vaginal margins or vaginal invasion within 0.5 cm of the surgical margin.

### Data collection and follow-up

From the medical records, we retrieved the clinicopathologic information of the patients, such as baseline demographics, histologic type, FIGO stage, tumor size, surgical approach, pelvic lymph node involvement, risk factors, and adjuvant treatments.

Follow-up information was obtained through outpatient clinic appointments and a telephone questionnaire. Patient data were censored at the time of last follow-up or cancer-related death. The primary outcome was DFS, defined as the period from surgery to the detection of recurrence or CC-related death. The secondary outcome was OS, defined as the period from initial surgery to all-cause death.

### Statistical analysis

SPSS version 22 was used to conduct all statistical analyses. For continuous variables, unpaired Student’s *t* test or Mann-Whitney *U* test were used. For categorical variables, Pearson’s chi-squared test or Fisher’s exact test were used. To compare and analyze survival data between the two surgery groups, Kaplan-Meier curves and log-rank tests were utilized. Clinical risk factors affecting survival outcomes were analyzed using Cox regression models. The threshold for statistical significance was fixed at *P*<0.05.

## Results

### Patient characteristics

**Fig. 1:**
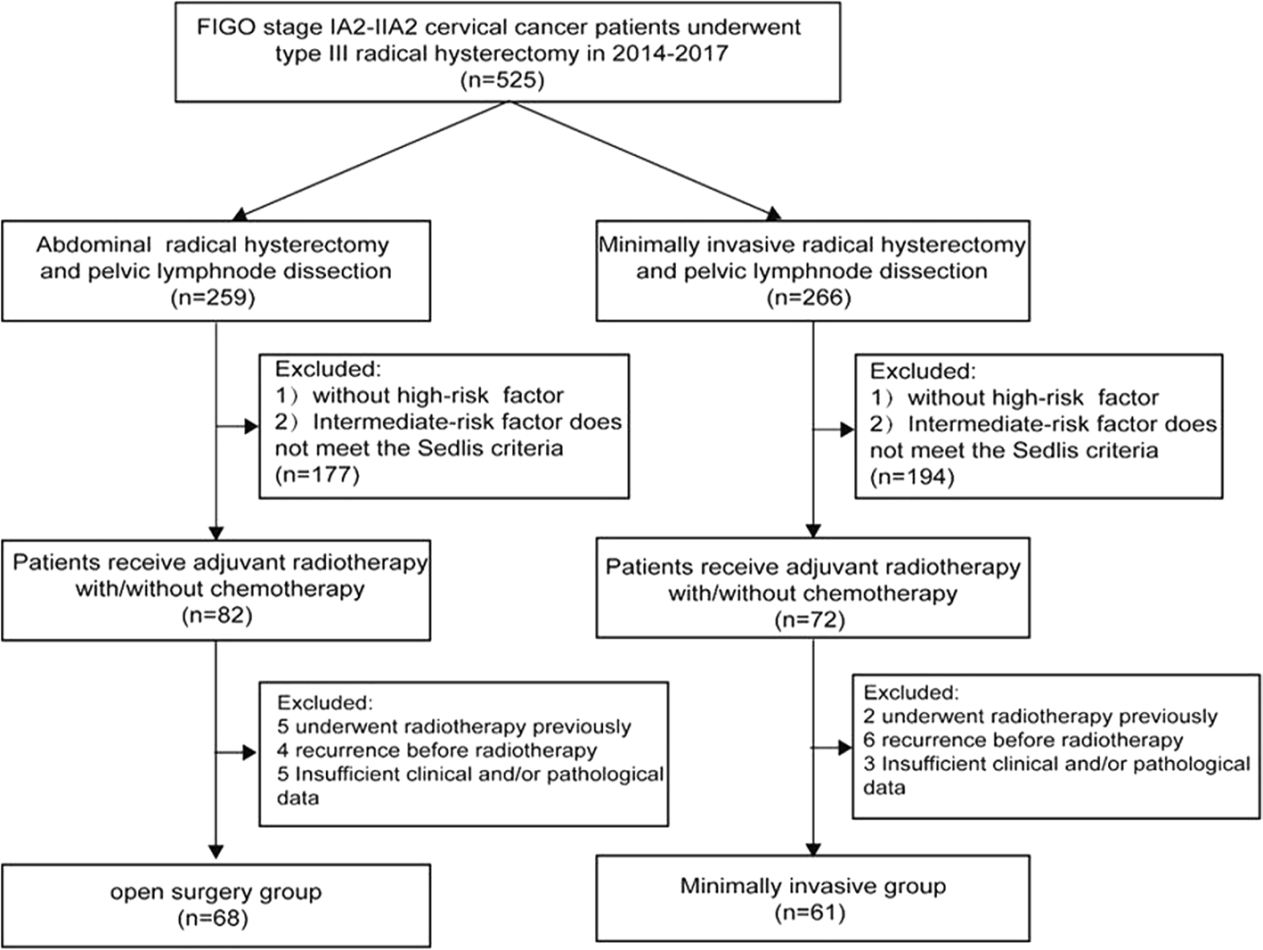
Flow chart for the sample selection

A total of 129 patients with IB1-IIA2 CC were enrolled in the study, including 68 (52.7%) in the open surgery group and 61 (47.2%) in the MIS group (Fig. 1). Table 1 summarized the clinicopathological information. Except for tumor stage and the presence of LVSI, there were no significant differences in age, tumor size, tumor histology, tumor differentiation, pelvic lymph node, stromal invasion, surgical margin, and prognostic risk between the two groups. For FIGO tumor staging, the most prevalent stage for the MIS group was stage IB1 (45.9%) and IIA1 (47.1%) for the open surgery group (*P*=0.046). For LVSI, the proportion of patients with LVSI was higher in the MIS group compare with the open surgery group (42.6% vs*.* 19.1%. *P*=0.004).

**Table 1:** Baseline characteristics

Characteristic	Open surgery (***n***=68)	MIS (***n***=61)	***P*** value
Age, years	46 (27–61)	47 (35–59)	0.4
FIGO stage			0.046
IB1	24 (35.3)	28 (45.9)	
IB2	5 (7.4)	12 (19.7)	
IIA1	32 (47.1)	17 (27.9)	
IIA2	7 (10.3)	4 (6.6)	
Tumor size			0.097
>4.0cm	11 (16.2)	14 (23)	
>2cm, ≤4cm	44 (64.7)	28 (45.9)	
≤2.0cm	13 (19.1)	19 (31.1)	
Histology			0.510
Squamous cell carcinoma	62 (91.2)	58 (95.1)	
Adenocarcinoma	4 (5.9)	1 (1.6)	
Others	2 (2.9)	2 (3.3)	
Tumor differentiation			0.09
1	3 (4.4)	2 (3.3)	
2	45 (66.2)	32 (52.5)	
3	20 (29.4)	27 (44.3)	
Pelvic lymph node			0.053
Positive	20 (29.4)	28 (45.9)	
Negative	48 (70.6)	33 (54.1)	
LVSI			0.004
Positive	13 (19.1)	26 (42.6)	
Negative	55 (80.9)	35 (57.4)	
Stromal invasion			0.753
Invasion depth>1/2	58 (85.3)	47 (77.0)	
Invasion depth<1/2	10 (14.7)	12 (19.7)	
No	0 (0)	2 (3.3)	
Surgical margin			0.212
Positive	5 (7.4)	1 (1.6)	
Negative	63 (92.6)	60 (98.4)	
Prognostic risk group			0.053
High-risk	21 (30.9)	29 (47.5)	
Intermediate-risk	47 (69.1)	32 (52.5)	

*FIGO* International Federation of Gynecology and Obstetrics, *LVSI* lymph vascular space invasion

### Treatment

Table 2 summarized the postoperative adjuvant therapy protocol. Gynecologists and radiation oncologists administered the treatments. Overall, the two groups had equivalent rates of postoperative RT, CT, and intracavitary radiotherapy (*P*>0.05). The most common radiation therapy in the study was IMRT (open surgery group vs*.* MIS group, 76.5% vs*.* 83.6%), with few patients receiving 3D-CRT (open surgery group vs*.* MIS group, 23.5% vs*.* 16.4%). Besides, most patients were treated with postoperative SCRT (open surgery group vs*.* and MIS group, 88.2% vs*.* 88.5%), and only a few patients received postoperative RT alone (open surgery group vs*.* and MIS group, 11.8% vs*.* 11.5%). Despite equal postoperative adjuvant treatments, the MIS group had a shorter median time interval (TI) from surgery to CT (7 days vs*.* 8 days, *P*=0.014) and from surgery to RT (28 days vs*.* 35 days, *P*<0.001) compared with the open surgery group. Few patients who suffered from grade 3 or 4 gastrointestinal (GI) or genitourinary (GU) toxicities and hematologic (HT) toxicity were the most common severe side effect (open surgery group vs. MIS group, 47.1% vs. 26.2%; *P*=0.015), and detailed information about adverse events during treatment and follow-up time was recorded in Supplementary Table 1.

**Table 2:** Treatment details for two groups

Treatment	Open surgery (***n***=68)	MIS (***n***=61)	***P*** value
Technique			0.313
IMRT	52 (76.5)	51 (83.6)	
3D-CRT	16 (23.5)	10 (16.4)	
Postoperative treatment			0.959
RT alone	8 (11.8)	7 (11.5)	
RT+CT	60 (88.2)	54 (88.5)	
Intracavitary radiotherapy	7 (10.4)	3 (5.0)	0.332
Chemotherapy before RT			0.242
Yes	53 (77.9)	42 (68.9)	
No	15 (22.1)	19 (31.1)	
CT cycles before RT	1 (0–3)	1 (0–4)	0.1
TI (surgery to CT), days	8 (5–17)	7 (5–14)	0.014
TI (surgery to RT), days	35 (18–100)	28 (16–120)	<0.001
≤42	45 (66.2)	51 (83.6)	
43–49	13 (19.1)	3 (4.9)	
50–56	4 (5.9)	1 (1.6)	
57–63	0 (0.0)	1 (1.6)	
≥64	6 (8.8)	5 (8.2)	
Total CT cycles	3.5 (0–6)	4 (0–6)	0.089
Grade 3–4 adverse effect			
Hematologic	32 (47.1)	16 (26.2)	0.015
Gastrointestinal	2 (2.9)	0 (0)	0.506
Genitourinary	4 (5.9)	2 (3.3)	0.683

*IMRT* intensity-modulated radiation therapy, *3D-CRT* three-dimensional conformation radiotherapy, *RT* radiotherapy, *CT* chemotherapy, *TI* time interval

### Survival outcomes

The last follow-up was in April 2021 and the average follow-up duration was 67.5 months (interquartile range: 52–78 months). Six patients in the open surgery group and 8 patients in the MIS group were lost to follow-up.

**Fig. 2:**
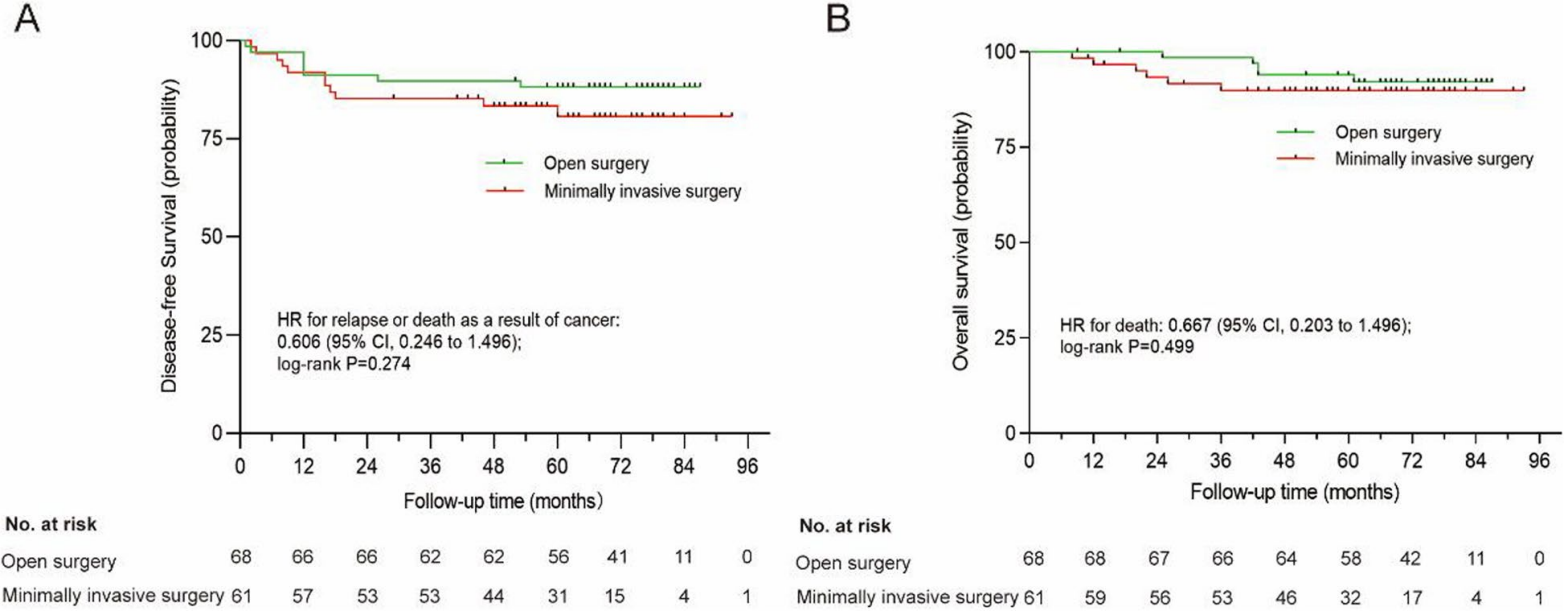
Comparisons of survival outcomes of early-stage patients with intermediate- or high-risk factors in the MIS group and open surgery group. **A** Disease-free survival (DFS). **B** Overall survival (OS)

**Fig. 3:**
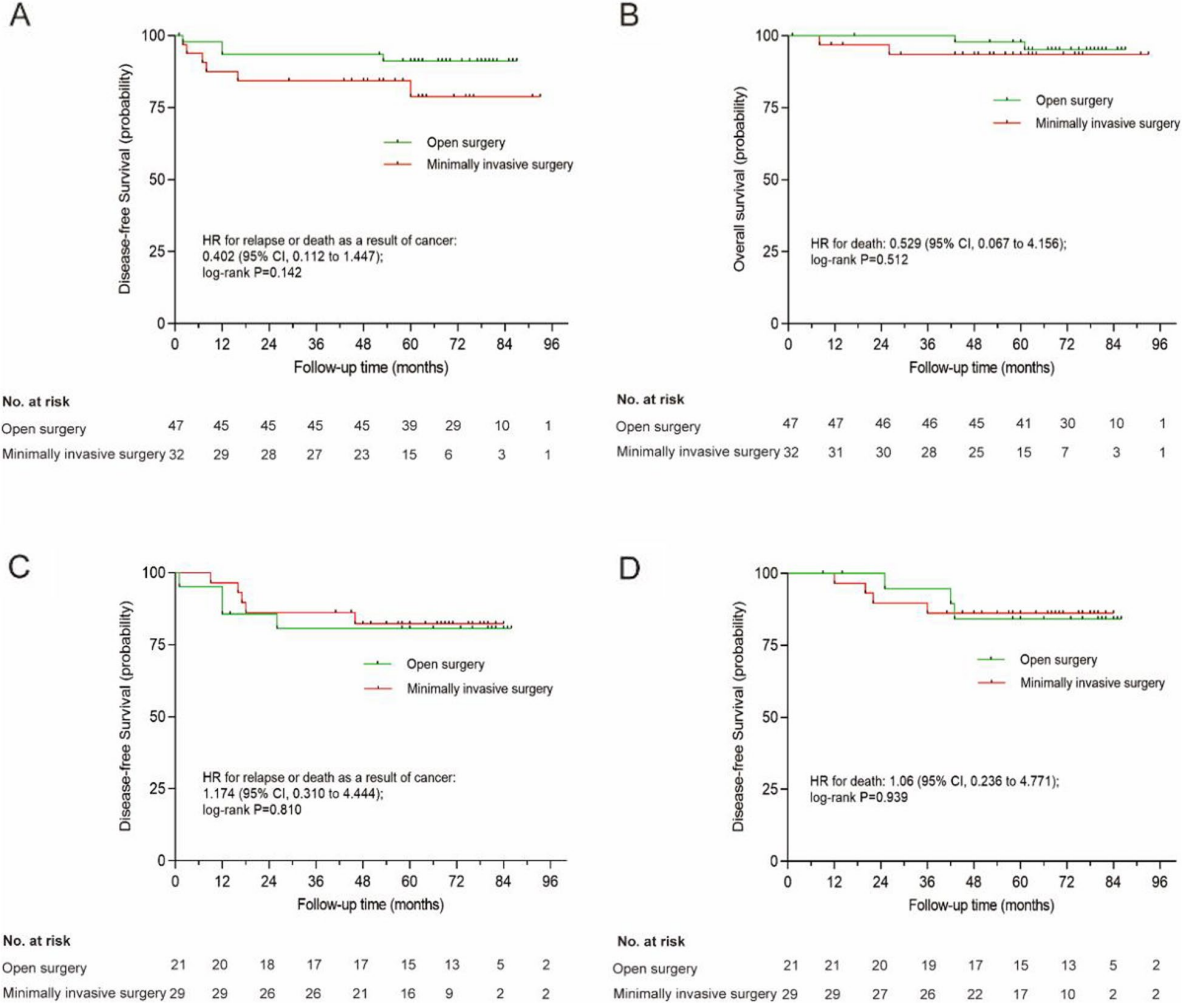
Comparisons of survival outcomes of early-stage patients with intermediate- or high-risk factors in the MIS group and open surgery group. **A** Disease-free survival (DFS) in intermediate-risk cervical cancer patients. **B** Overall survival (OS) in intermediate-risk cervical cancer patients. **C** Disease-free survival (DFS) in high-risk cervical cancer patients. **D** Overall survival (OS) in high-risk cervical cancer patients

Patients in the MIS group who underwent postoperative RT or CRT had a slightly lower 3-year DFS and OS than those in the open surgery group, but there was no statistical difference (85.2% vs. 89.7%, *P*=0.274; 89.9% vs. 98.5%, *P*=0.499; Fig. 2A, B). Subgroup survival analyses of the intermediate-risk and high-risk groups revealed no significant differences in DFS and OS between the two surgical approaches (Fig. 3A, D). Further, we analyzed the prognostic factors of DFS, which was shown in Table 3. In the univariate analysis, FIGO stage, tumor size, and TI from surgery to RT (>7 weeks) were significantly associated with DFS. In the multivariate analysis, independent variables predicting poor DFS outcomes were tumor size (>4 cm) and TI from surgery to RT (>7 weeks).

**Table 3:** Univariate and multivariate analysis for DFS

Characteristics	Univariate analysis			Multivariate analysis		
	HR	95%CI	***P*** value	HR	95% CI	***P*** value
Age (≥45 vs*.*<45)	1.16	0.47–2.88	0.75			
FIGO stage	1.79	1.11–2.87	0.016			
Tumor size (>4cm vs*.* ≤4cm)	4.33	1.76–10.67	0.001	4.42	1.79–10.92	0.001
Histology (SC vs*.* others)	0.66	0.15–2.84	0.57			
Differentiation	0.97	0.42–2.25	0.94			
Deep invasion	0.62	0.22–1.72	0.36			
Surgical margin	1.13	0.15–8.47	0.91			
LVSI	1.15	0.44–3.02	0.78			
LN metastasis	1.22	0.49–3.04	0.67			
TI (surgery to CT) (>7 days vs*.* ≤7 days)	0.77	0.25–2.39	0.65			
TI (surgery to RT) (>7 weeks vs*.* ≤7 weeks)	4.22	1.60–11.22	0.004	4.34	1.64–11.50	0.003
CT cycles (>4)	2.01	0.82–4.94	0.13			

*SC* squamous cancer, *LVSI* lymph vascular space invasion, *LN* lymph node, *TI* time interval, *CT* chemotherapy, *RT* radiotherapy

The recurrence and mortality rates were summarized in Table 4. During the follow-up time, 8 (11.8%) patients in the open surgery group and 11 (18%) patients in the MIS group experienced tumor recurrence. The recurrence in open surgery group includes local (*n* = 5, 62.5%) and distant areas outside of the pelvis (*n* = 3, 37.5%). The local and distant recurrence for the MIS group were 5 (45.5%) and 6 (54.5%), respectively. There was no difference in the recurrence rate or pattern between the two groups (*P*=0.463 and *P*=0.709). Before the last follow-up, 5 (7.3%) patients in the open surgery group and 6 (9.8%) patients in the MIS group had died of CC (*P*=0.614).

**Table 4:** Recurrences and death

Characteristics	Open surgery (***n***, %)	MIS (***n***, %)	***P*** value
Patients with recurrences	8 (11.8)	11 (18)	0.463
Recurrence site			0.709
Local	5 (62.5)	5 (45.5)	
Vagina	4	4	
Pelvis	1	1	
Distal	3 (37.5)	6 (54.5)	
Lung	1	2	
Multi-recurrence	1	2	
Unknown	1	2	
Total death	5 (7.3)	6 (9.8)	0.614

## Discussion

Korean Gynecologic Oncology Group found that in low-risk early-stage CC patients, laparoscopic group had lower DFS, but no significant difference in OS [17]. Worse survival outcomes were demonstrated in patients receiving MIS by LACC trial; however, this trial did not include patients who met the criteria for adjuvant therapy but did not receive it [8]. Another study with a patient cohort similar to the LACC trial suggested that the disparate outcomes between MIS and open surgery may be explained by discrepancies in compliance with adjuvant therapy [4]. Based on the above findings, we analyzed all patients with intermediate- or high-risk who met the criteria for adjuvant therapy after receiving MIS and open surgery for CC at our institution.

Our study showed that the DFS and OS times were similar between the MIS and open surgery groups, contrasting with the outcomes of the LACC trial. The results may differ due to several factors. Firstly, the surgical-related factors that result in poor patient survival, such as utilizing a uterine manipulator, the effect of insufflation gas (CO_2_), and the degree of resection, can be improved by postoperative RT or CRT [18–20]. Secondly, many studies indicated that a longer adjuvant RT wait-time after RH result in poorer oncologic outcome in early-stage CC [21, 22]. In LACC trial, there was no significant between-group difference in the time to initiation of any adjuvant therapy. While in our study, the TI from surgery to postoperative CT and RT were shorter in the MIS group, resulting in a shorter overall treatment time, which was a critical factor for pelvic control and survival in CC [23]. In addition, most of our patients received sequential CRT (SCRT), which was similar with the SCRT group reported by Huang et al. [15]. They concluded that compared with RT or CRT, SCRT improved the DFS and decreased the distant recurrence, as well as the risk of death. One of their limitations was not stratifying laparotomy or laparoscopy for randomization. In our study, we stratified the patients by surgical approaches, MIS group had a shorter median TI from surgery to CT and from surgery to RT compared with the open surgery group, and the DFS and OS had no significant difference in two groups. In the current clinical practice, adjuvant CCRT for early-stage CC is usually allowed to initiate 4–6 weeks after RH, providing time for wound healing. In our study, the timely initiation of the adjuvant radiotherapy in the majority of patients confirmed the safety of early administration of adjuvant CT, which was consisting with previous study [24]. DeBoer et al. also supported a role for temporizing CT if delays to CRT are anticipated [25].

Most patients in our study received pelvic IMRT, which could reduce the toxicity of postoperative RT with a non-inferior survival outcome [26, 27]. In GOG-92, a randomized trial of postoperative RT versus no further therapy for stage IB CC after RH, the results revealed that the 3-year PFS and OS were around 86% and 88%, respectively [28]. In high-risk patients, the 3-year PFS and OS were around 84% and 88%, respectively [12]. Similarly, our research demonstrated that the 3-year DFS and OS rates in open surgery versus MIS groups were 89.7% vs*.* 85.2% and 98.5% vs*.* 89.9%, respectively. Meanwhile, IMRT reduced GI and GU toxicity, with a greater incidence of grade 3 or higher acute HT complications [29].

For early-stage CC, short TI from surgery to adjuvant CRT had better OS and DFS than long-interval patients, and TI was an independent predictor of DFS and local recurrence-free survival [22, 30]. In our study, cox-multivariate analyses showed that TI from surgery to RT beyond 7 weeks was adverse independent prognostic factors for DFS. Consistent with our results, Hanprasertpong et al. [30] found that delaying adjuvant therapy in patients with early-stage CC beyond 4 weeks after surgery resulted in lower recurrence-free survival. Tumor size at diagnosis represents the key element in order to offer a tailored treatment not only aiming to the best oncological outcomes but also to the best quality of life. Available data suggested that tumor size affected tumor treatment outcomes [31]. Di Donato et al. found that women with high tumor grade, aggressive histology, and tumor size ≥5 cm have a higher risk of recurrence [32]. Interestingly, we also found that tumor size >4 cm was an independent risk factor for DFS. Thus, for patients with a tumor size>4 cm, MIS and open surgery should be proposed only warning patients on the higher recurrence rate.

Our study had some limitations. First, it was a retrospective study, which might have potential confounding biases, such as the selection bias introduced by physicians in determining which patients should be considered for minimally invasive radical hysterectomy versus abdominal hysterectomy. The gynecologists are cline to perform MIS on patients with early FIGO stages and small tumors. Second, the sample size was insufficient to found the significant difference, we need a larger sample size to conform the difference. On the other hand, the DFS and OS had no significant difference in MIS and ORH groups, and we wonder what the result would have been if there was a third arm with no treatment after surgery. More clinical trials to testing these hypotheses are warranted.

## Conclusions

Based on the population we studied, for early-stage CC patients with intermediate- or high-risk factors who received postoperative adjuvant SCRT, although the difference was not significant, the DFS and OS in the MIS group were slightly lower than the ORH group, and the delayed RT more than 7 weeks and tumor size >4 cm were the risk factors for recurrence.

## Supplementary Information

**Additional file 1: Supplementary Table 1.** Adverse events reported during treatment and follow-up time.

## Data Availability

The datasets used and/or analyzed during the current study are available from the corresponding author upon reasonable request.

## Abbreviations

CC: Cervical cancer
CRT: Chemoradiation
MIS: Minimally invasive surgery
OS: Overall survival
DFS: Disease-free survival
LACC: Laparoscopic Approach to Carcinoma of the Cervix
RT: Radiotherapy
LVSI: Lymph vascular space invasion
EBRT: External beam radiotherapy;
CT: Chemotherapy
IMRT: Intensity-modulated radiotherapy
3D-CRT: Three-dimensional conformal radiation therapy
TI: Time interval
GI: Gastrointestinal
GU: Genitourinary
HT: Hematologic
SCRT: Sequential CRT
