# Supplementary material for: The impacts of minimally invasive surgery on intermediate- or high-risk cervical cancer patients received adjuvant radiotherapy
Source: World J Surg Oncol. 2022 Nov 28;20:372. doi: 10.1186/s12957-022-02820-x (PMC9703778; doi:10.1186/s12957-022-02820-x)
Supplement: Supplementary file 1 — Additional file 1: Supplementary Table 1. Adverse events reported during treatment and follow-up time. [file 12957_2022_2820_MOESM1_ESM.docx]

**Supplementary Table 1** Adverse events reported during treatment and follow-up time

| Acute and late toxicities | Open surgery group (n=68) | | MIS group (n=61) | |
| --- | --- | --- | --- | --- |
|  | All grades | Grade 3/4 | All grades | Grade 3/4 |
| Hematologic |  |  |  |  |
| Anemia | 27 (39.7) | 17 (25) | 18 (29.5) | 5 (8.2) |
| Neutropenia | 20 (29.4) | 13 (19.2) | 25 (41) | 10 (16.4) |
| Thrombocytopenia | 5 (7.3) | 2 (2.9) | 3 (4.9) | 1 (1.6) |
| Gastrointestinal (GI) |  |  |  |  |
| GI bleeding | 5 (7.3) | 1 (1.5) | 7 (11.5) | 0 |
| Diarrhea | 7 (10.3) | 0 | 5 (8.2) | 0 |
| Abdominal pain | 11 (16.2) | 0 | 3 (4.9) | 0 |
| [Rectovaginal fistula](javascript:;) | 1 (1.5) | 1 (1.5) | 0 | 0 |
| Genitourinary (GU) |  |  |  |  |
| Dysuria | 5 (7.3) | 1 (1.5) | 6 (9.8) | 0 |
| Radiation cystitis | 8 (11.8) | 3 (4.4) | 5 (8.2) | 2 (3.3) |
